# Supplementary material for: Low Levels of IgM and IgA Recognizing Acetylated C1-Inhibitor Peptides Are Associated with Systemic Lupus Erythematosus in Taiwanese Women
Source: Molecules. 2019 Apr 26;24(9):1645. doi: 10.3390/molecules24091645 (PMC6539680; doi:10.3390/molecules24091645)
Supplement: Supplementary file 1 [file molecules-24-01645-s001.zip › 2 Supplementary Figure 1 Ac-protein Adducts v1.1.pdf]

Acetylation-protein Adducts

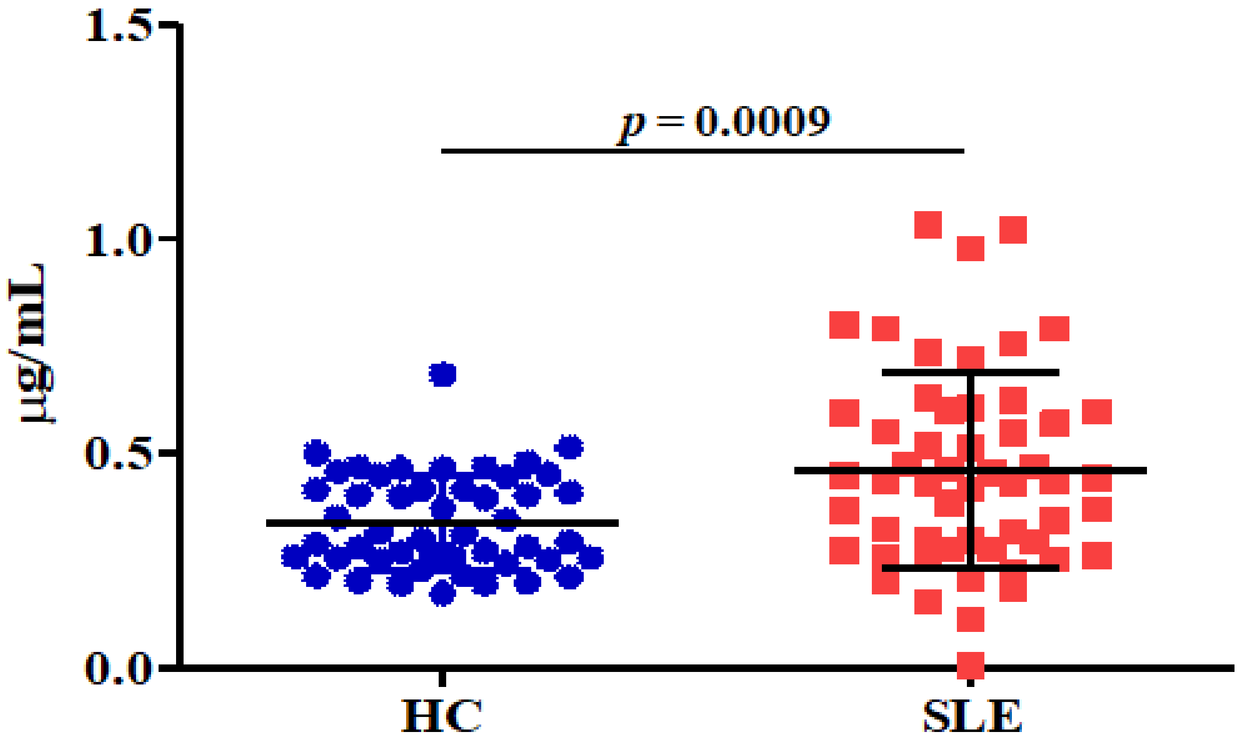

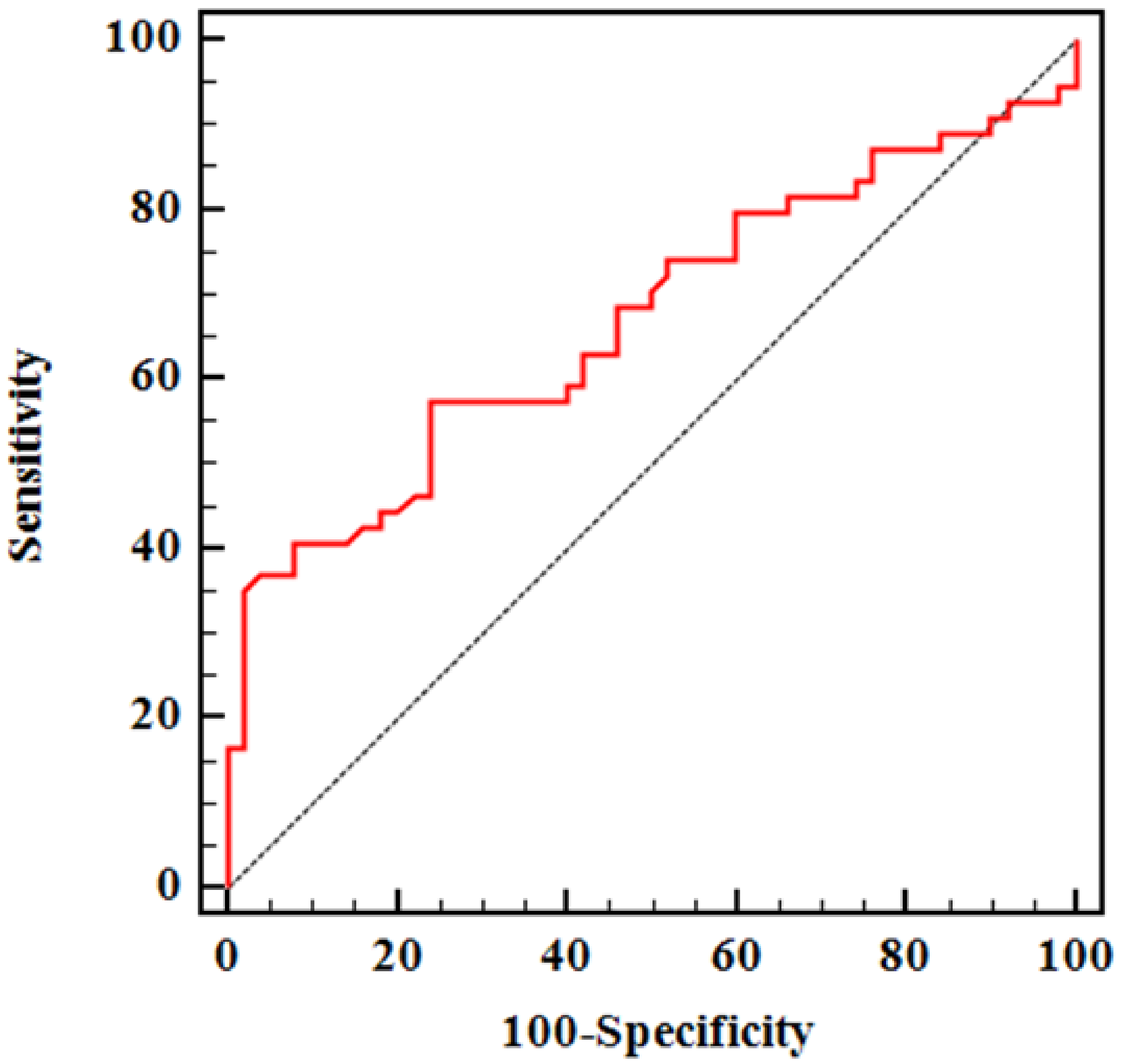

HC = 0.34 ± 0.11
SLE = 0.46 ± 0.22

	AUC (95% CI)	Sensitivity (95% CI)	Specificity (95% CI)
Acetylation-protein Adducts	0.67 (0.57, 0.76)	70.4% (56.4, 82.0)	50.0% (35.5, 64.5)

Cut-off value: 0.299
