# Supplementary material for: Low Levels of IgM and IgA Recognizing Acetylated C1-Inhibitor Peptides Are Associated with Systemic Lupus Erythematosus in Taiwanese Women
Source: Molecules. 2019 Apr 26;24(9):1645. doi: 10.3390/molecules24091645 (PMC6539680; doi:10.3390/molecules24091645)
Supplement: Supplementary file 1 [file molecules-24-01645-s001.zip › 2 Supplementary information 20181021 v1.0.pdf]

Supplementary Information

1. Supplementary Methods

1.1. Measurement of Serum Protein Concentrations

Serum protein concentrations were quantified using a Pierce™ Coomassie Plus (Bradford) Assay Kit (Thermo Scientific, Waltham, MA, USA). Briefly, serum samples (4 μL) and bovine serum albumin (BSA) standards were added to a 96-well plate containing a reagent (200 μL) and incubated for 10 min at 37°C. The optical density was determined at 595 nm using an enzyme-linked immunosorbent assay. Protein concentrations of the samples were calculated according to the BSA standard concentrations (0–1500 μg/mL).

1.2. One-Dimensional Sodium Dodecylsulfate Polyacrylamide Gel Electrophoresis and In-Gel Digestion

Serum protein samples (50 μg) were run on an 8% sodium dodecylsulfate polyacrylamide gel electrophoresis (SDS-PAGE) (Hoefer®, Holliston, MA, USA) gel. The entire gel was stained with a Coomassie brilliant blue (CBB) staining solution (Bio-Rad Laboratories, Hercules, CA, USA). Gel slices were cut according to molecular weights of 96–105 kDa. The gel slices were repeatedly destained using 25 mM NH_4HCO_3 and 50% (v/v) acetonitrile (1:1). After drying, the gel slices were incubated with 2% β-mercaptoethanol and 25 mM NH_4HCO_3 for 20 min at room temperature in the dark to reduce disulfide bonds in the proteins. In the cysteine alkylation process, 10% 4-vinylpyridine in 25 mM NH_4HCO_3 and 50% acetonitrile were added and incubated for 20 min. The gel slices were soaked in 25 mM NH_4HCO_3 for 10 min, and they were dried in a Speed-Vac (Thermo Electron, Waltham, MA, USA) for 20 min. Subsequently, modified trypsin (100 ng, Promega, Mannheim, Germany) in 25 mM NH_4HCO_3 was added to the gel slices, and the

mixture was incubated at 37℃ overnight after tryptic digestion. Tryptic peptides were collected and dried in a Speed-Vac. Tryptic peptides were frozen at −20℃ until further analysis. Tryptic peptides were dissolved in 0.1% formic acid before use.

1.3. Post-Translational Modification Identified Using Nano-Liquid Chromatography-Tandem Mass Spectrometry

The mobile phases of 0.1% formic acid in water (solvent A) and 0.1% formic acid in acetonitrile (solvent B) were run at a flow rate of 0.5 μL/min using a 30-min linear gradient of 5%–35% solvent B followed by 95% solvent B for a duration of 10 min. After each full scan (m/z range of 200–2000), a data-dependent acquired tandem mass spectrometry (MS/MS) scan for a series of precursor ions was chosen on the basis of conventional MS spectra (Survey Scan) that were triggered at high resolution (M/DM, 60,000 full-width half-maximum). The acquired the spectrum for fragmented ions produced by collision-induced dissociation, whereas the latter inspected the accurate mass and charge state of the selected precursor ions. An MS/MS dataset was analyzed using Xcalibur 2.0 SR1 software (Thermo Electron) to yield peak lists. Peptide sequences were identified through ion scans obtained from MS/MS data against a human protein sequence database with 157,433 entries (obtained from UniProt; http://www.uniprot.org/, 2016/11) by using the SEQUEST algorithm. The database search allowed fixed modifications of cysteine residues (S-pyridylethylation, 105.057849 Da), variable modifications of methionine residues (oxidation, 15.994915 Da), and the peptide mass tolerance at 50 ppm and fragment mass tolerance at 1 Da. These peptides were filtered using Xcorr versus the charge state with a statistically significant level of $p < 0.05$. Xcorr was used for a match with 1.9 for a charge state of +1, 2.2 for a charge state of +2, and 3.75 for a charge state of +3. SEQUEST results required a delta correlation value of ≤0.1. Protein identification required at least two

unique peptides to match, and the Xcorr score of each peptide was set to >2.5. Subsequently, the post-translational modification (PTM) of the C1-inhibitor (C1-INH) was analyzed using our PTM finder-in-house program to identify PTM peptide sequences and PTM sites. Precursor ions were monoisotopic masses, and the analytical error was set to <20 ppm. All modified MS_1 spectra were manually confirmed, and fragmented ions were labeled as b, y, y-NH_3, and b-H_2O ions.

1.4. Immunoprecipitation

For the immunoprecipitation (IP) process, 2 μg of the mouse anti-C1-INH monoclonal antibody (M01, Abnova, New Taipei City, Taiwan) was bound to 2 mg Protein A Sepharose™ CL-4B (GE Healthcare Bio-Sciences, Pittsburgh, PA, USA) in 400 μL NET-2 buffer (50 mM Tris-HCl, 150 mM NaCl, and 0.5% triton X100, pH 7.4) and then gently mixed with 100 μg of IgG-removal serum proteins for 2 h at 4°C. Beads were centrifuged thrice with 1 mL of NET-2* buffer (50 mM Tris-HCl, 150 mM NaCl, and 0.05% triton X100, pH 7.4) at 4000 rpm for 3 min. Subsequently, the C1-INH was immunoprecipitated through an 8% SDS-PAGE gel and Western blotting using a rabbit polyclonal acetylated-lysine antibody.

1.5. Western Blotting

Serum proteins (2 μg in 6% gel) or the immunoprecipitated C1-INH (100 μg in 8% gel) was separated in SDS-PAGE gels and then transferred to a polyvinylidene difluoride membrane (GE Healthcare Life Sciences, Piscataway, NJ, USA) using transfer buffer (25 mM Tris base, 192 mM glycine, and 20% methanol, pH 8.0). The membrane was blocked with protein-free F1 1 Blocking Buffer (LIONBIO, New Taipei City, Taiwan), followed by incubation with a mouse anti-C1-INH monoclonal antibody (dilution 1:20,000, M01, Abnova Corporation). Acetylation (Ac)

modification of the C1-INH was detected using a rabbit polyclonal acetylated-lysine antibody (dilution 1:10,000, #9441 Cell Signaling Technology, Danvers, MA, USA). This antibody was then reacted with a horseradish peroxidase (HRP)-conjugated goat antimouse IgG antibody (dilution 1:15,000, sc-2055, Santa Cruz Biotechnology, Dallas, TX, USA) or an HRP-conjugated rabbit antigoat IgG antibody (dilution 1:20,000, sc-2922, Santa Cruz Biotechnology) as the secondary antibody after washing. Reactive protein bands were detected using the Luminata™ Forte Western HRP Substrate (Millipore, Billerica, MA, USA). The acquired band intensity was imaged using an ImageQuant 400™ Imager (GE Healthcare Life Sciences) and ImageJ software (National Institutes of Health, Bethesda, MD, USA). The SDS-PAGE gel was stained with CBB (Bio-Rad Laboratories, Hercules, CA, USA) as the loading control.

1.6. Measurement of Autoantibody Isotypes against C1-INH$^{367-385}$ and C1-INH$^{367-385}$ Ac Peptides

A 96-well microplate was coated with peptides (phosphate-buffered saline [PBS] at pH 7.0) at 10 μg/mL and incubated overnight at 4°C. After the plate was washed and blocked, 100-fold diluted serum (PBS at pH 7.0) was loaded onto the plate and incubated at 37°C for 2 h. Subsequently, rabbit antihuman IgM-HRP (dilution 1:10^4, Santa Cruz Biotechnology), rabbit antihuman IgG-HRP (dilution 1:10^4, Santa Cruz Biotechnology), or goat antihuman IgA-HRP (dilution 1:10^4, Novus Biologicals, Littleton, CO, USA) was added to detect the deposition of autoantibodies against the C1-INH$^{367-385}$ and C1-INH$^{367-385}$ Ac peptides at 37°C for 1 h. The HRP-conjugated antibody was reacted with BD OptEIA™ TMB Substrate Reagent Set (BD Biosciences, San Diego, CA, USA) for 10 min at room temperature. The reaction was stopped by adding 1 N HCl.

1.7. Determination of Serum Ac-Protein Adducts

Serum samples (100 µL; 10 µg/mL) or acetylated BSA (Sigma-Aldrich, St. Louis, MO, USA) standards (0–10 µg/mL) were coated on a 96-well plate and incubated at 37°C for 2 h. The plate was washed with PBS and blocked with PBS containing 3% BSA (Sigma-Aldrich) at room temperature for 1 h. A rabbit polyclonal acetylated-lysine antibody (dilution 1:10,000, #9441 Cell Signaling Technology) was then added, and the samples and standards were incubated at 37°C for 1 h. Next, an HRP-conjugated goat anti-rabbit IgG antibody (sc-2054; dilution 1:20,000; Santa Cruz Biotechnology) was added at 37°C for 1 h. The bound antibody-HRP reacted with the BD OptEIA™ TMB Substrate Reagent Set (BD Biosciences) for 10 min at room temperature. The reaction was stopped by adding 1 N HCl. The absorbance was measured at 450 nm with the reference standard at 620 nm. Concentrations of serum Ac-protein adducts were calculated according to a standard acetylated BSA curve. Concentrations of Ac-protein adducts are expressed in micrograms per milliliter (µg/mL).
