# Supplementary material for: Low Levels of IgM and IgA Recognizing Acetylated C1-Inhibitor Peptides Are Associated with Systemic Lupus Erythematosus in Taiwanese Women
Source: Molecules. 2019 Apr 26;24(9):1645. doi: 10.3390/molecules24091645 (PMC6539680; doi:10.3390/molecules24091645)
Supplement: Supplementary file 1 [file molecules-24-01645-s001.zip › 2 Supplementary Table 2 20181021 v1.0.pdf]

Supplementary Table 2

Characteristic	**HC [a]** (n = 50)	**SLE [b]** (n = 54)	**RA [b]** (n = 40)
**Age [c]**	42.00 ± 8.41	41.17 ± 11.65	54.85 ± 10.66
**Gender**			
Female	50	54	40
**Disease duration (years)**	N.A.[d]	7.13 ± 7.00	N.A.
**SLEDAI**	N.A.	8.88 ± 5.54	N.A.
**Current therapy**			
NSAIDs (%)	N.A.	27.80	N.A.
DMARDs (%)	N.A.	94.40	N.A.
Other (e.g. steroids) (%)	N.A.	68.52	N.A.
**Clinical tests**			
Anti-ds DNA - positive (%)	N.A.	52.80	0.0
Anti-Sm - positive (%)	N.A.	20.00	0.0
ANA - positive (%)	0.0	82.61	47.5
RF - positive (%)	0.0	7.69	72.5
Anti-Ro (SSA) - positive (%)	0.0	46.00	5.0
Anti-La (SSB) – positive (%)	0.0	16.00	0.0
CRP – positive (%)	N.A.	25.00	37.5
ESR – positive (%)	N.A.	69.23	87.5

a. HCs were tested by Union Clinical Laboratory in Taiwan: Rheumatoid factor (Latex RF reagent, Siemens Medical Solutions Diagnostics, USA; cutoff of < 15.0 IU/mL), ANA (FLUORO HEPANA Test, Medical & Biological Laboratories, Japan), SSA (BioPlex 2200 ANA, Bio-Rad Laboratories, Hercules, CQA, USA; cutoff of < 100 AU/mL), and SSB (BioPlex 2200 ANA, Bio-Rad Laboratories; cutoff of < 100 AU/mL).

b. Patient samples were analyzed at the Department of Laboratory Medicine, Shuang-Ho Hospital, Taiwan: Rheumatoid factor (Latex RF reagent, Siemens Medical Solutions Diagnostics; cutoff of < 15.0 IU/mL), ANA (FLUORO HEPANA Test, Medical & Biological Laboratories, Japan), SSA (EliA™ Ro FLUOROENZYMEIMMUNOASSAY, Phadia GmbH, Freiburg, Germany; cutoff of < 7.0 IU/mL), and SSB (EliA™ La FLUOROENZYMEIMMUNOASSAY, Phadia GmbH; cutoff of < 7.0 IU/mL), anti-dsDNA:(EliA™ ds DNA FLUOROENZYMEIMMUNOASSAY, Phadia GmbH, Freiburg, Germany; cutoff of < 10 IU/mL), anti-Sm:(EliA™ SmD[P] FLUOROENZYMEIMMUNOASSAY, Phadia GmbH, Freiburg, Germany; cutoff of < 7.0 IU/mL), CRP (CardioPhase® hsCRP, SIEMENS, Munich, Germany; cutoff of < 0.5 mg/dL) and ESR (VACUETTE® , Greiner Bio-One, Kremsmünster, Austria; cutoff of < 10 mm/1 hr).

c. HC vs. RA, $p < 0.0001$; SLE vs. RA, $p < 0.0001$.

d. N.A., Not available.
