# Supplementary figures and images for: Low Levels of IgM and IgA Recognizing Acetylated C1-Inhibitor Peptides Are Associated with Systemic Lupus Erythematosus in Taiwanese Women
Source: Molecules. 2019 Apr 26;24(9):1645. doi: 10.3390/molecules24091645 (PMC6539680; doi:10.3390/molecules24091645)

**Serum samples: 54 SLE, 40 RA and 50 HCs**

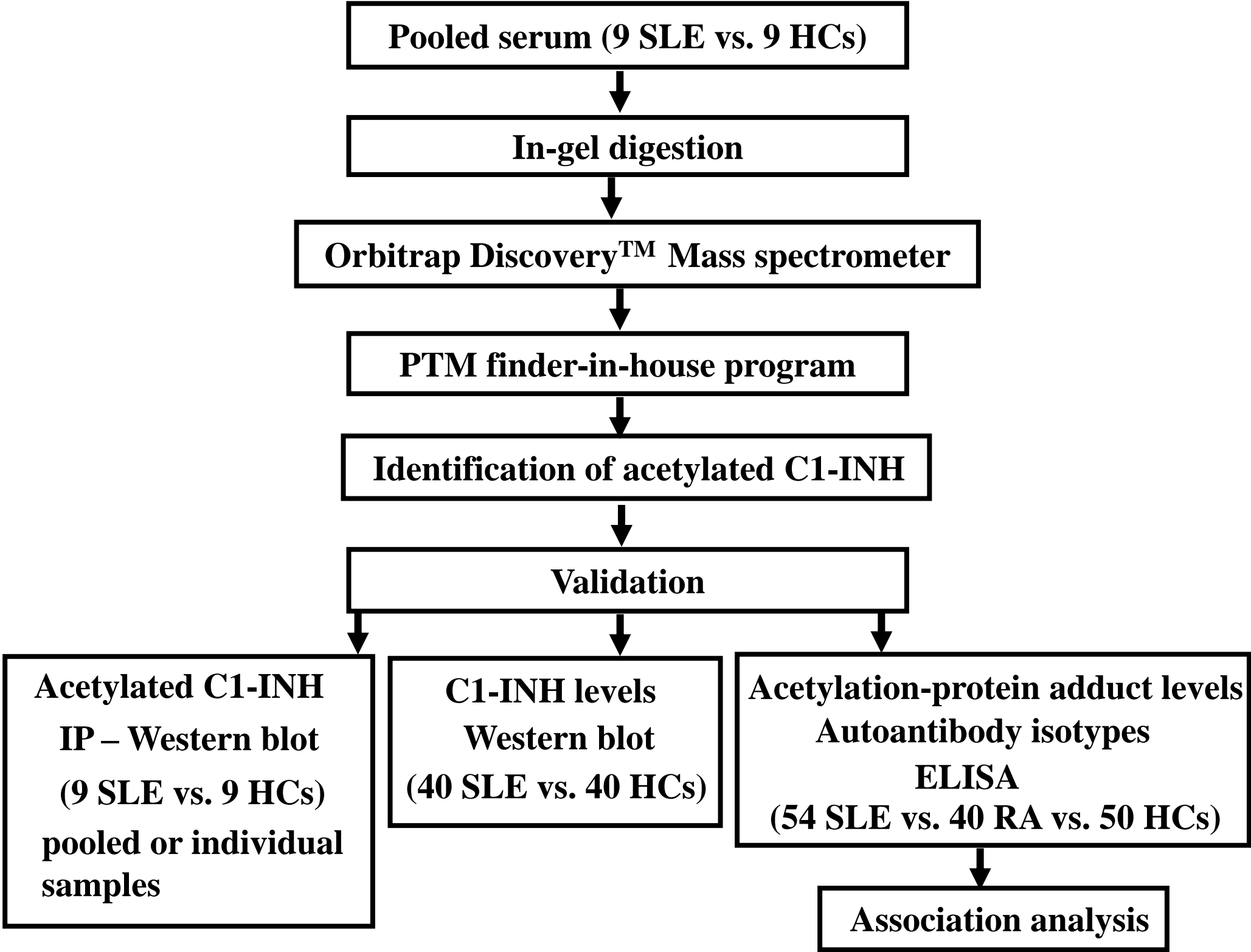

## **Supplementary Figure 1 Flow chart.**

Supplement: Supplementary file 1 [file molecules-24-01645-s001.zip › 2 Supplementary Figure 1 Flow chart.pdf]
